# Prevalence of germline mutations in the *TP53* gene in patients with early-onset breast cancer in the Mexican population

**DOI:** 10.1186/s12885-019-5312-2

**Published:** 2019-02-01

**Authors:** Lenny N. Gallardo-Alvarado, María Teresa Tusié-Luna, María Isabel Tussié-Luna, José Díaz-Chávez, Yayoi X. Segura, Enrique Bargallo-Rocha, Cynthia Villarreal, Luis A. Herrera-Montalvo, Enrique M. Herrera-Medina, David F. Cantu-de Leon

**Affiliations:** 1 Instituto Nacional de Cancerología, San Fernando Avenue #22, Zip Code 14080 Tlalpan, Mexico City Mexico; 2 Unidad de Biología Molecular y Medicina Genómica. Instituto Nacional de Ciencias Médicas y Nutrición Salvador Zubirán, Instituto de Investigaciones Biómédicas de la UNAM, Vasco de Quiroga #15, Zip Code 14080 Tlalpan, Mexico City Mexico; 3 División de Investigación, Facultad de Medicina, Universidad Nacional Autónoma de México (UNAM), Av. Universidad #3000. Col. Universidad Nacional Autónoma de México, C.U., Zip Code 04510 Coyoacán, Mexico City Mexico; 4 Unidad de Investigación en Virología y Cáncer, Hospital Infantil de México Federico Gómez, Dr. Márquez No. 162, Col. Doctores, Zip Code 06720 Cuauhtémoc, Mexico City Mexico; 5 Facultad de Ciencias, Universidad Nacional Autónoma de México (UNAM), Av. Universidad #3000. Col. Universidad Nacional Autónoma de México. C.U., Zip Code 04510 Mexico City, Mexico

**Keywords:** Breast cancer, Li-Fraumeni, Young women

## Abstract

**Background:**

Heterozygous germline *TP53* gene mutations result in Li-Fraumeni Syndrome (LFS). Breast cancer (BC) is the most frequent tumor in young women with LFS. An important issue related to BC in the Mexican population is the average age at diagnosis, which is approximately 11 years younger than that of patients in the United States (U.S.) and Europe. The aim of this study was to determine the prevalence of germline mutations in *TP53* among young Mexican BC patients.

**Methods:**

We searched for germline mutations in the *TP53* gene using targeted next-generation sequencing (NGS) in 78 BC patients younger than 45 years old (yo) who tested negative for *BRCA1/2* mutations. A group of 509 Mexican women aged 45yo or older without personal or family BC history (parents/grandparents) was used as a control.

**Results:**

We identified five patients with pathogenic variants in the *TP53* gene, equivalent to 6.4% (5/78). Among patients diagnosed at age 36 or younger, 9.4% (5/55) had pathogenic *TP53* mutations. Three of these variants were missense mutations (c.844C > T, c.517G > A, and c.604C > T), and the other two mutations were frameshifts (c.291delC and c.273dupC) and had not been reported previously. We also identified a variant of uncertain clinical significance (VUS), c.672G > A, which causes a putative splice donor site mutation. All patients with *TP53* mutations had high-grade and HER2-positive tumors. None of the 509 patients in the healthy control group had mutations in *TP53.*

**Conclusions:**

Among Mexican BC patients diagnosed at a young age, we identified a high proportion with germline mutations in the *TP53* gene. All patients with the *TP53* mutations had a family history suggestive of LFS. To establish the clinical significance of the VUS found, additional studies are needed. Pathogenic variants of *TP53* may explain a substantial fraction of BC in young women in the Mexican population. Importantly, none of these mutations or other pathological variants in *TP53* were found in the healthy control group.

## Background

Breast cancer (BC) is the leading cancer-related cause of death in women between the ages of 20 and 59 years [1]. The general population has an 8 to 12% lifetime risk of developing BC [2, 3]. This percentage significantly increases when there is family cancer history associated with a hereditary cancer predisposition syndrome [4–10]. With the use of next-generation sequencing (NGS), many genes (> 60) associated with the risk of hereditary cancer have been identified [4, 11–14].

Germline *TP53* mutations cause Li-Fraumeni Syndrome (LFS) (Online Mendelian Inheritance in Man [OMIM] #151623) [15]; patients with this syndrome show a predisposition for a broad spectrum of tumors, including adrenocortical carcinoma, bone and soft-tissue sarcomas, brain tumors, and early-onset BC [16]. The lifetime risk of developing cancer in patients with LFS is > 90% by the age of 60. This percentage differs depending on gender, at approximately 68% in males and 93% in females. This difference is due to the high rate of women with LFS who develop BC at an early age (pre-menopause), making BC the most common cancer in LFS carriers (24–31%) [3, 16–20]. The most commonly used criteria to facilitate the diagnosis of LFS are those characterized by Chompret et al., 2001. Recently, other researchers have revised these criteria by adding other factors that include more individuals at risk of having *TP53* mutations.

The *TP53* gene, located at chromosome 17p13.1, is a tumor suppressor gene that is 20 kilobases (kb) long and contains 11 exons. It encodes the transcription factor P53, which plays major roles in both cell growth regulation and maintenance of cellular homeostasis. In response to cellular stress, the p53 protein regulates target gene expression to induce cell cycle arrest, apoptosis, senescence, DNA repair, or changes in metabolism. The activity of p53 is ubiquitously lost in human cancers either by mutation of the *TP53* gene itself or by the loss of cell signaling upstream or downstream of p53 [21, 22]. Among the high-penetrance genes associated with inherited BC syndromes, mutations in *BRCA1* and *BRCA2* are the most prevalent and have been found in 20–40% of hereditary BC cases [12]. Recent studies have suggested that mutations in *TP53* are responsible for 1% of BCs and between 15 and 20% of all hereditary cancers [3, 17, 23, 24]. *TP53* gene mutations are present in 2–6% of BC patients younger than 35 [25–28]. Other genes related to BC risk are *PTEN*, *TP53, CHEK2*, *ATM*, *STK11/LKB1*, *CDH1*, *RAD50, PALB2* and others [24, 29].

Mexico has a complex population structure including indigenous populations, European (especially Spanish) populations and African immigrants. The percentages vary widely across the country [30]. At present, the majority of the Mexican population is mestizo with similar proportions of European and Native American ancestries, with up to 5% black ancestry in individuals living on the coasts [31]. The average age of BC diagnosis in the Mexican population is approximately 11 years younger than in other populations (particularly those of the U.S. and Europe) (50 years old in Mexico vs. 61 years old in the U.S. and Europe) [32, 33]. A study performed in the U.S. indicates that the differences in age at presentation of BC can be accounted for by the ancestry of the population: 47% of Hispano-American patients had BC before the age of 50, while only 25% of Caucasian patients had BC before that age. This observation is highly relevant to the search for germline mutations in cancer-predisposing genes in women with BC in Mexico because, as mentioned previously, the principal characteristic of cancer-predisposing syndromes is the early age of onset [34].

In Mexican women with BC, the prevalence of *BRCA1/2* mutations is approximately 15%. The Mexican founder mutation (*BRCA1* ex9-12del) accounts for 30% of *BRCA*-associated BC. Despite the high prevalence of mutations in *BRCA 1/2* genes, a large proportion of the young population with BC does not have variations in those genes [34, 35].

The objective of this study was to determine the prevalence of germline mutations in the *TP53* gene among Mexican women with early-onset BC.

## Methods

We performed a descriptive study in young women with BC who were diagnosed and treated at the Instituto Nacional de Cancerología (INCan) in México City from December 2013 to October 2015. The Institutional Ethics Committee approved the study. After obtaining a signed informed consent, we did an interview to obtain personal and family history. Tumor data were extracted from the pathology reports in the patients’ clinical file, and DNA was obtained from blood lymphocytes.

### Study population

In a cohort of patients at high risk for hereditary BC, there were 394 women younger than 45 years old. From this group, 65 were identified as *BRCA* (+), 32 had *BRCA* VUS, and 279 were negative for the *BRCA* mutation. From the group of *BRCA*-negative patients, the first 78 consecutive patients were selected for this evaluation. A group of 509 Mexican women aged 45 years or older without personal or family BC history (parents/grandparents) was used as a control. This group is part of a Mexican-mestizo population-based cohort, for whom whole-exome sequence data were available (SIGMA Type 2 Diabetes Consortium, 2014).

### Molecular testing

#### Library construction, target gene capture, and massively parallel sequencing

Two hundred nanograms of each genomic DNA sample was divided into eight different restriction reactions. After digestions were completed, the fragmented gDNAs were hybridized to custom-design biotinylated HaloPlex probes directed to the complete genomic regions of *BRCA1, BRCA2,* and *TP53*. This resulted in a total of 253,521 nt of targeted DNA in the presence of a unique 8-bp index primer cassette, which allowed the multiplexing of samples. Hybridization resulted in circularization of gDNA fragments and incorporation of the indexes and Illumina sequencing motifs. DNA probe biotinylation allowed the capture of target DNA hybrids using streptavidin-coated magnetic beads. After ligation, the eluted targeted fragments were amplified by PCR (18–19 cycles) to produce a target-enriched sample. Libraries were purified using AMPure XP beads (Beckman Coulter, CA). Enrichment of each library was validated using the 2100 Bioanalyzer (Agilent Technologies) and Qubit quantification.

DNA sequencing was performed on a HiSeq2500 sequencing system (Illumina, Inc., San Diego, CA) with 2 x 150pb paired-end reads using v3 reagents following the manufacturer’s instructions. The average fraction of on-target reads was 0.38. The minimum average range suitable for the panel was established at 2000x. Up to 20 samples were multiplexed in one lane (average of 3500x coverage after removal of duplicate reads). Our platform detected single nucleotide variants (SNV), insertions/deletions (indels), copy number variants (CNVs) and gene rearrangements, including a *BRCA1* del [9–12] mutation described as a founder mutation in BC Mexican patients [33].

All mutations were validated by PCR amplification and Sanger sequencing. Patients received genetic counseling before and after the test.

### Data analysis

Pipeline data analysis was performed as described by Cabanillas et al., 2017 [36]. Briefly, FASTQ files were evaluated using quality checks from Fast QC (http://www.bioinformatics.babraham.ac.uk/projects/fastqc/). Trimmomatic was used to find and remove low-quality bases and contaminants of adapters and sequencing indexes [37]. Each library was then aligned to the human genome data (hg19 / GRCh37) as the reference. Processing and alignment were carried out through the BWA program and SAMtools (http://sourceforge.net/projects/samtools/files), respectively [38].

Germline mutations were identified using VarScan2 tools (http://sourceforge.net/projects/varscan). Identified variants were annotated using several databases including Ensembl, CCDS, RefSeq, Pfam, dbSNP, 1000 Genomes, COSMIC, ICGC and HGMD and by using different scoring algorithms for functional prediction such as SIFT, Polyphen, Mutation Assessor, Mutation Taster, FATHMM, and FATHMM-MKL.

In addition, allelic frequencies of the identified *TP53* mutations were analyzed in a control group of 509 Mexican women aged 45 years or older than without personal or family BC history, for whom whole-exome sequence data were available [39].

## Results

**Fig. 1:**
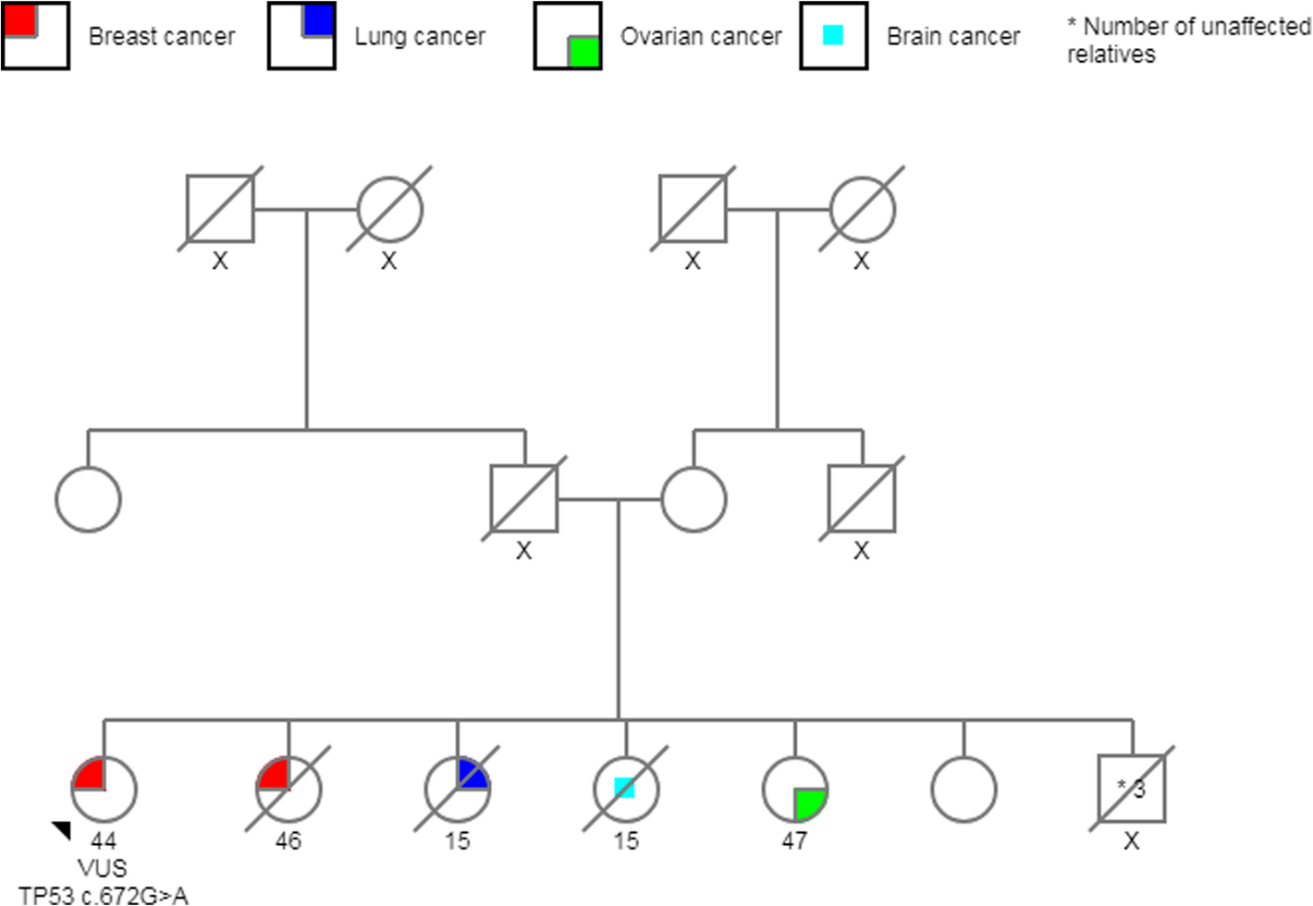
Pedigree *TP53* c.672G > A (VUS). Patient with BC diagnosed in the breast at age 44; one sister died of BC at 46 years of age, another sister died at age 15 from lung cancer, another sister died at 15 years of age from brain cancer, and a sister was diagnosed with ovarian cancer at age 47

Five pathogenic *TP53* variants were identified according to the variant classification criteria described by Cabanillas et al., 2017 [36]. The overall prevalence of these variants was 6.4% (5/78), a percentage that rises to 9.4% if we consider only patients who were diagnosed before the age of 36 (5/53). In addition, we identified a VUS, *c.672G > A*, that caused a silent mutation in a splice donor site in a patient who met the Chompret criteria for LFS (Fig. 1). None of the 509 patients in the healthy control group had mutations in *TP53.*

These mutations were identified from a group of 78 patients with early-onset BC who were found to be negative for mutations in *BRCA1* and *BRCA2* through HISPANEL mutation screening [33]. The median age at diagnosis was 32 years, > 50% (*n* = 41) presented with locally advanced stages or with metastatic disease, and the prevalent histologic types included invasive ductal and lobular carcinomas (CCI and CLI) (Table 1).

**Table 1:** Distribution of patients according to clinical characteristics

Characteristic	Cases (*n* = 78)	
	N	%
Age^a^	32 (23–43)	
Clinical Stage		
I/IIA	31	39.8
IIB/III/IV	41	52.5
Not specified	6	7.7
Histological Type		
IDC/ILC	68	87.2
DCIS/LCIS	4	5.1
Others	1	1.3
Not specified	5	6.4
Histological Subtype		
Luminal A/B	29	37.2
HER2 +	13	16.7
Triple Positive	11	14.1
Triple Negative	19	24.4
Not specified	6	7.7

^a^ Mean (max-min) IDC: infiltrate ductal carcinoma, ILC: infiltrate lobular carcinoma, DCIS: ductal carcinoma in situ, LCIS: lobular carcinoma in situ

We obtained a complete pedigree from all patients; 63 had BC and familial history of neoplasia in first-, second- and third-degree relatives, and 15 patients diagnosed with invasive breast cancer under the age of 45 years had no reported family history of cancer. All the Li-Fraumeni patients had a family history consistent with a high risk of hereditary predisposition to cancer syndrome, and among non-carrier patients, nearly 80% had a family history of cancer.

**Fig. 2:**
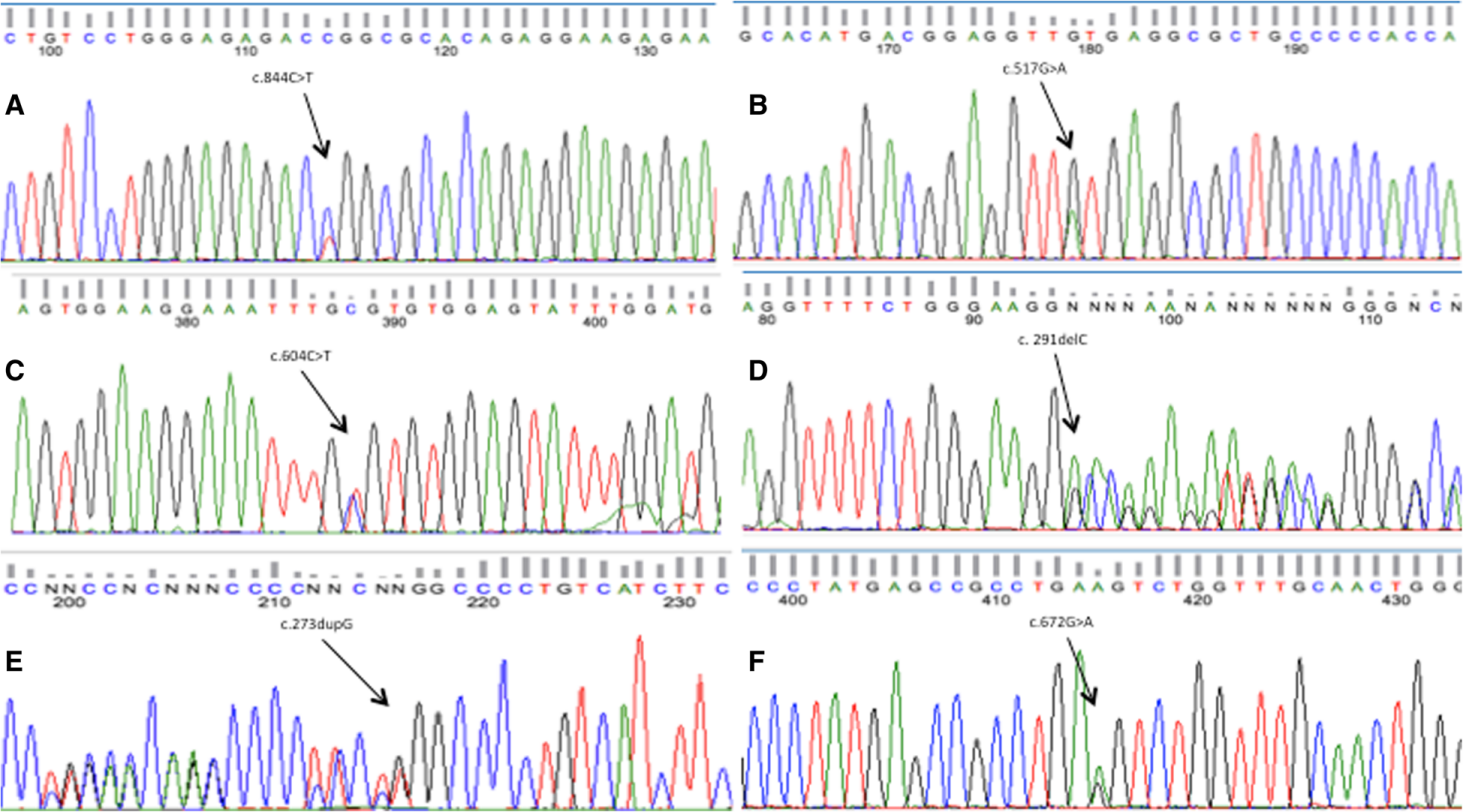
Pathogenic variants identified. Electropherograms showing heterozygous mutations in TP53. **a** TP53 c.844C>T, **b** TP53 c.517G>A, **c** TP53 c.604C>T, **d** TP53 c.291delC, **e** TP53 c.273dupG, **f** TP53 c.672G>A (VUS)

**Fig. 3:**
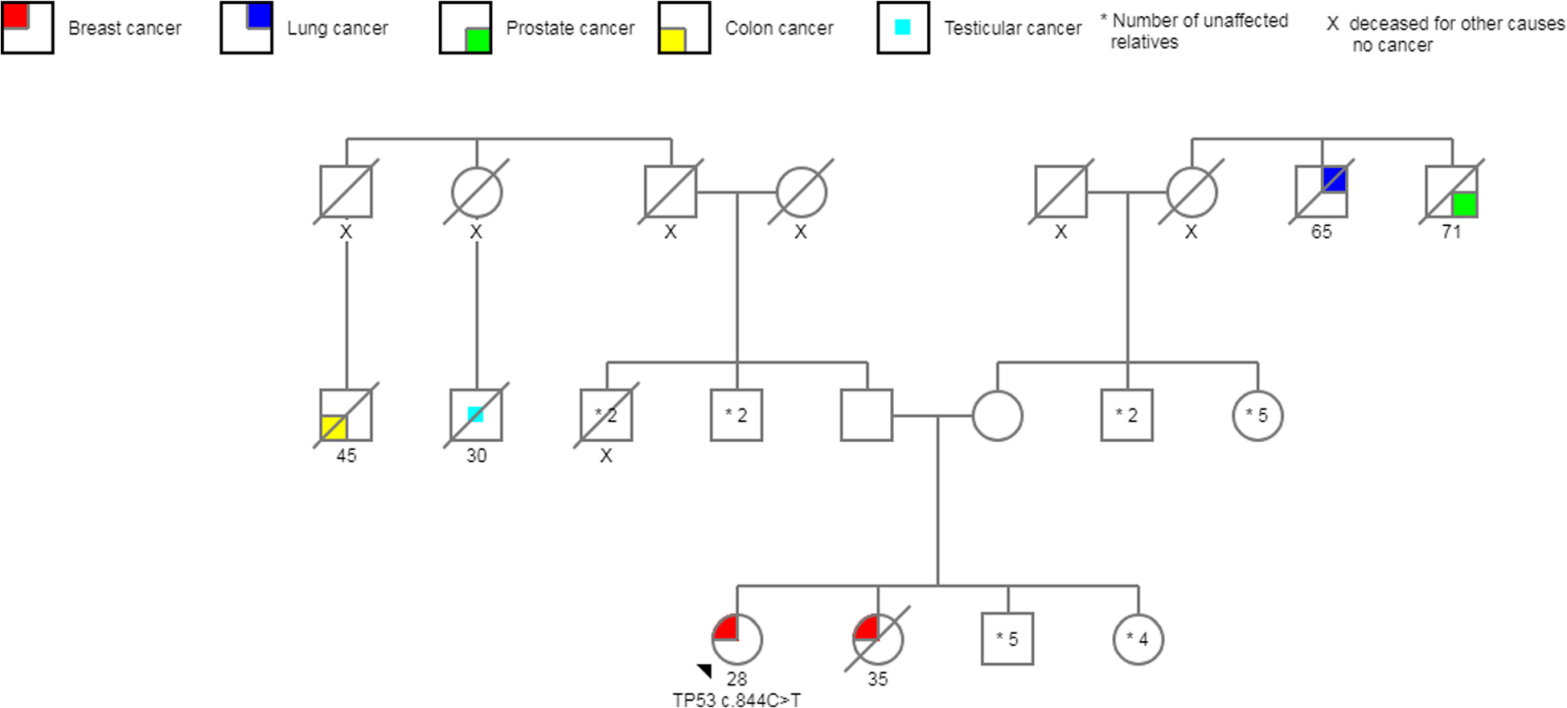
Pedigree *TP53* c.844C > T. The proband with BC diagnosed at age 26; her sister died of breast cancer at age 35, and there was a history of colorectal and testicular cancer on the paternal side, as well as lung and prostate cancer on the maternal side in third-degree relatives. Neither parents nor second-degree relatives are affected

**Fig. 4:**
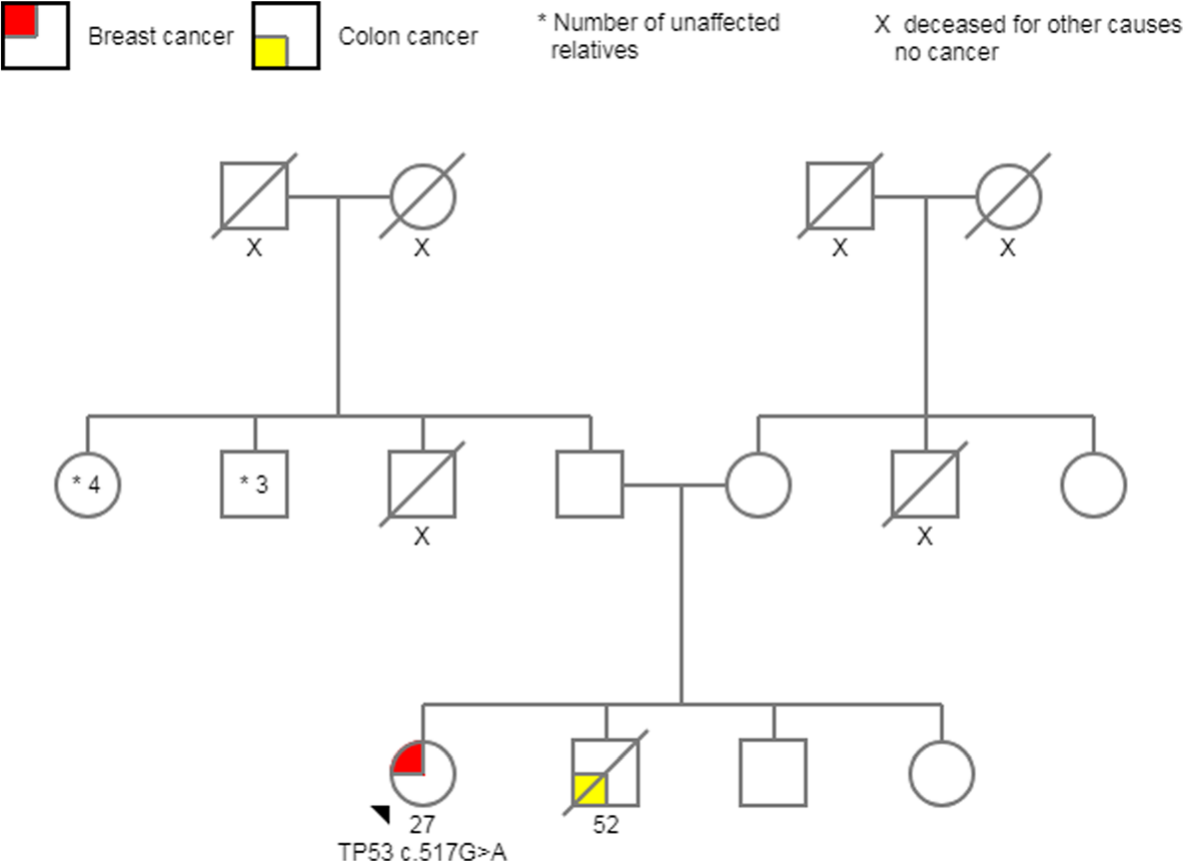
Pedigree *TP53* c.517G > A. The proband with breast cancer diagnosed at age 27, and one sibling died of colorectal cancer; the parents are unaffected

**Fig. 5:**
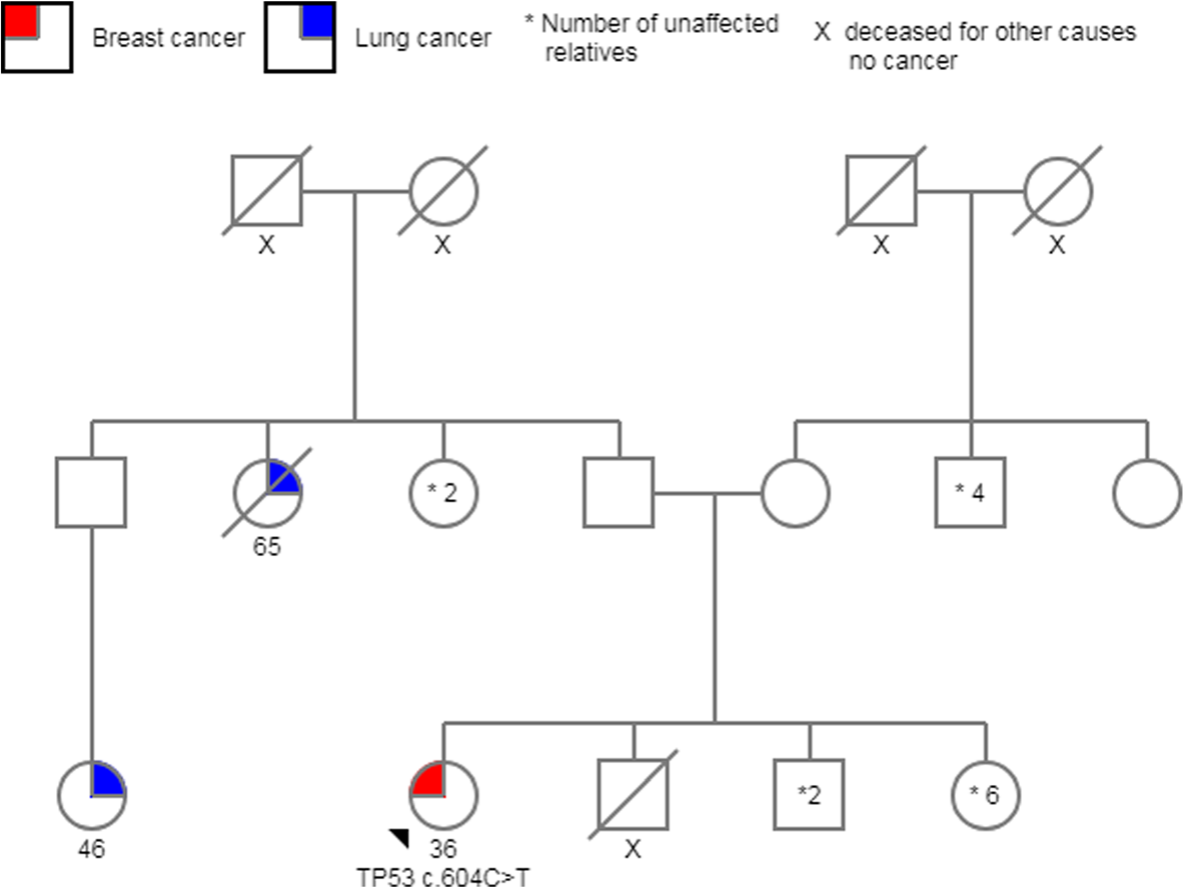
Pedigree *TP53* c.604C > T. Patient with BC diagnosed at age 36; parents and siblings are not affected. Lung cancer in an uncle and paternal cousin

**Fig. 6:**
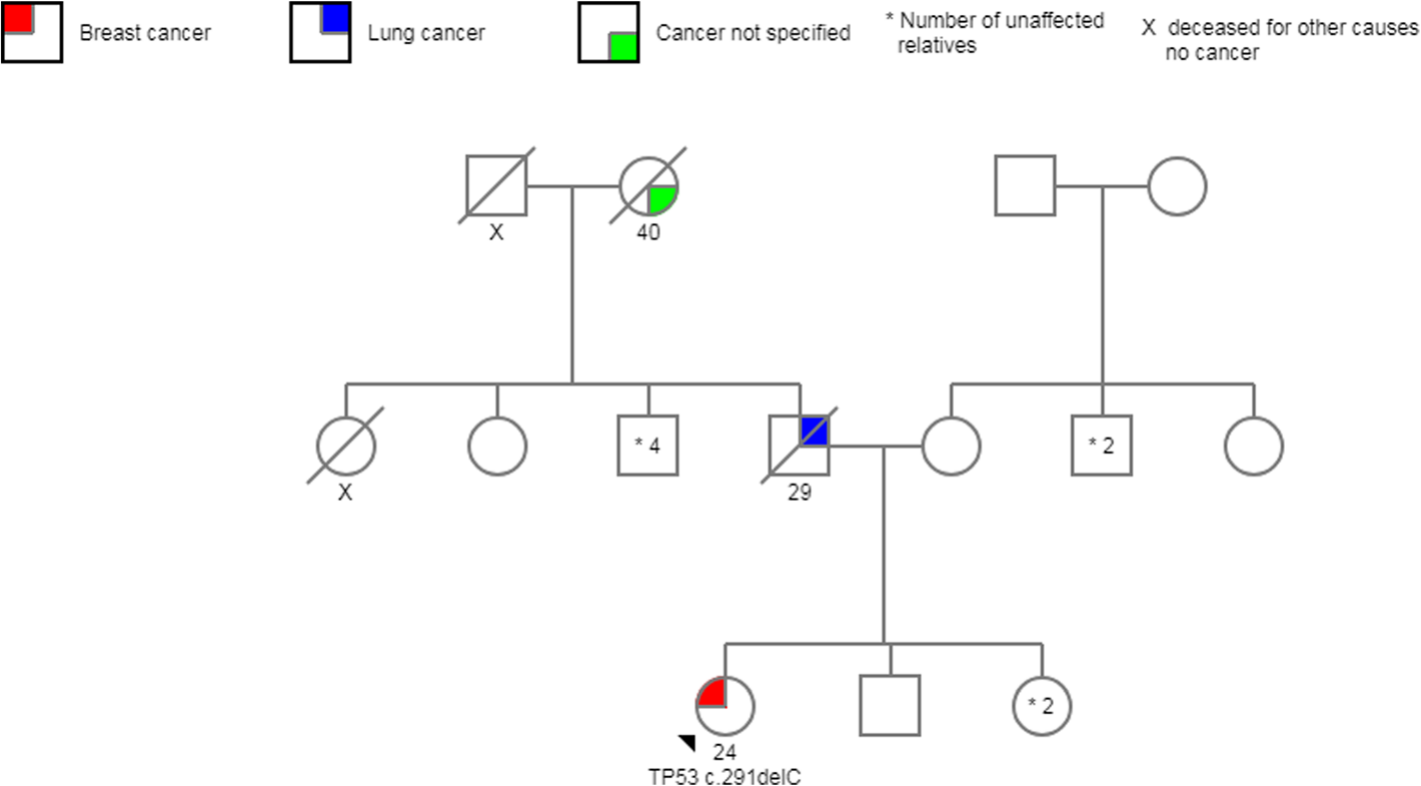
Pedigree *TP53* c.291delC. Patient with BC diagnosed at age 24, father died of lung cancer at 29 years of age, and paternal grandmother died at age 40 due to unspecified cancer

**Fig. 7:**
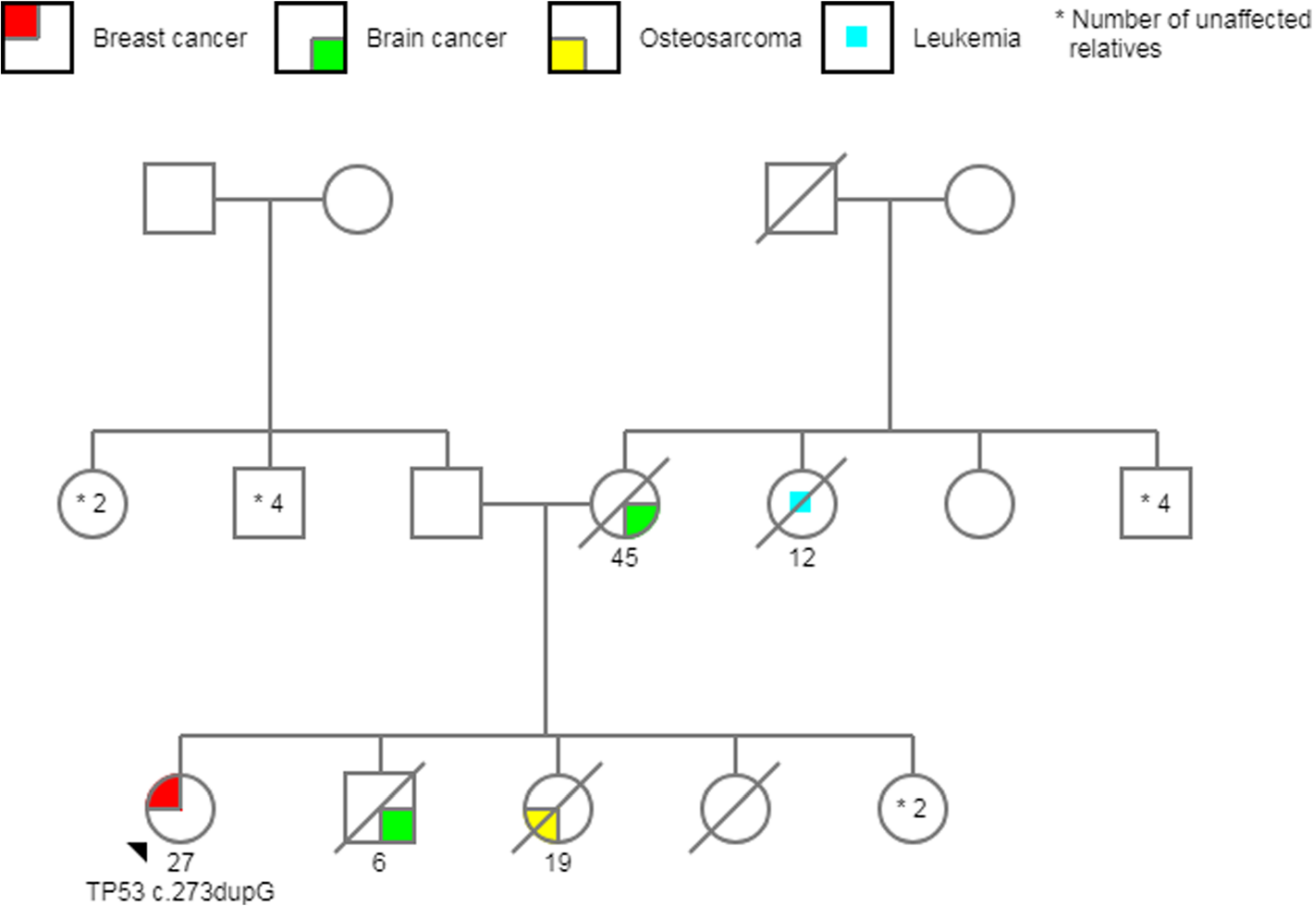
Pedigree *TP53* c.273dupG. A patient with a BC diagnosed at age 27, a brother who died at age six from a brain tumor, another sister who died at age 19 from osteosarcoma, a mother who died at 45 years of age due to a brain tumor, and a maternal uncle with leukemia at the age of 12

From *TP53-*negative patients with a positive family history of cancer, 19% had a first-degree relative affected by cancer, and the other 81% had at least one family member diagnosed with cancer before age 45 or two or more relatives with cancer at any age. Minimal and maximal coverage for the mutations detected in *TP53* were 843X and 2780X, respectively. Three of the pathogenic variants identified were missense mutations and had been reported to the International Agency for Research on Cancer (IARC) *TP53* database: *c.844C > T*; *c.517G > A*, and *c.604C > T*. The other two variants were frameshift mutations (*c.291delC* and *c.273dupC*) and had not been reported previously; however, they are likely to be pathogenic according to the biological consequence of this type of mutation (Fig. 2) (Table 2). All of these five mutations were found in BC women younger than 36 years old, and family history are shown in Figs. 3, 4, 5, 6 and 7. The last variant, *c.672G > A*, was classified as a VUS. It is found at a splice donor site and has a potential consequence for splicing (Figs. 1 and 2). None of these mutations were found among the 509 Mexican women aged 45 years or older without a personal or family BC history.

**Table 2:** Pathogenic mutation characteristics

	Exon	Nucleotide	Amino acid	Type of Mutation	Reported previously	Classification	Population
1	8	c.844C > T	p.R282W	missense	yes	pathogenic	Puebla
2	5	c.517G > A	p.V173 M	missense	yes	pathogenic	Oaxaca
3	6	c.604C > T	p.R202C	missense	yes	pathogenic	Morelos
4	4	c.291delC	p.P98Lfs*25	frameshift	no	pathogenic	San Luis Potosí
5	4	c.273dupG	p.P92Afs*57	frameshift	no	pathogenic	Morelos

All patients identified as having pathogenic variants in *TP53* had a familial history suggestive of LFS. The youngest age at diagnosis was 24, and the oldest age was 36. The clinical descriptions of the tumors are detailed in Table 3. The pedigrees of the mutation-positive probands show 16 cancer-affected first-, second-, and third-degree relatives. The most frequent tumor was lung cancer, followed by leukemia and colon cancer (Table 4). The HER2-positive patient with the VUS was 44 years old and pre-menopausal at the time of diagnosis with invasive ductal carcinoma (Fig. 2).

**Table 3:** Breast cancer characteristics in patients with *TP53* pathogenic mutations

Mutation	Diagnostic Age	CE*	Laterality	Histology	Estrogen Receptor	Progesterone Receptor	HER2
c.844C > T	28	IV	Right	ILC	–	–	+
c.517G > A	27	IIIA	Bilateral	IDC + DCIS	–	–	+
c.604C > T	36	IV	Left	ILC	+	+	+
c.291delC	24	IIB	Left	IDC	–	+	+
c.273dupG	27	IIB	Left	IDC	–	–	+

*ILC* invasive lobulillar carcinoma, *IDC* invasive ductal carcinoma, *DCIS* ductal carcinoma In situ

**Table 4:** Tumors present in the families of patients with mutations in *TP53*

Tumor	1st degree relatives	2nd degree relatives	3rd degree relatives	Total	Ranges of ages at diagnosis
Breast	1	0	0	1	< 35
Lung	1	2	1	4	25–70
Prostate	0	0	1	1	> 70
Testicular	0	0	1	1	< 30
Leukemia	0	1	1	2	10–30
CRC	1	0	1	2	40–60
Osteosarcoma	1	0	0	1	< 20
Brain	2	0	0	2	5–50
NS Tumor	0	1	0	1	< 40
Cervical	0	1	0	1	< 60

*NS* Not specified

## Discussion

The identification of hereditary syndromes that predispose carriers to develop early-onset BC is essential to consider in our population. In Mexican women with BC, the prevalence of *BRCA1/2* is approximately 15%. The Mexican founder mutation (*BRCA1* ex9-12del) accounts for 30% of *BRCA-*associated BC [35]. Despite the high prevalence of mutations in *BRCA*1/2 genes, a large proportion of the young population with BC did not present with pathogenic variants in these genes. The younger average age of diagnosis with BC in Mexican women might be explained in part by the age distribution of the population [31], but we must also consider hereditary syndromes that predispose carriers to develop early-onset BC other than *BRCA1/2* as another explanation for the young mean age of BC-onset in Mexico.

We found a high rate of mutations in *TP53* in women younger than 36 years old. The frequency in the present study is the second highest reported in the literature after that reported in Southern Brazil, where the founder mutation *TP53* (R337H) is known to be present in 12% of young women with BC [28, 40–46]. The first mutation identified, c.844C > T p.R282W, was initially reported as a founder mutation in the French-Canadian population [47]. The IARC database describes this mutation in 39 families [19, 46–48]. Our patient identified herself as a mestizo Mexicano, she and her family are from the center area of the country [47] (Fig. 3).

In the IARC database, the c.517G > A pathogenic variant is recorded in two Brazilian families and the other missense mutation c. 604C > T was registered only in Germany population [49]. Both of our patients denied that their ancestors come from another country. The last two mutations have not been described in the literature. Frameshift mutations account for 11% of all mutations found in the *TP53* database, and immunohistochemical analyses show a complete absence of TP53 protein expression [49] (Figs. 6 and 7).

Finally, a mutation classified as a VUS, localized at the end of exon 5 and likely to be impair *TP53* splicing, was found in a BC patient with a family history that meets the Chompret criteria [49] (Fig. 1). It is now well established that this kind of mutation can have critical effects on RNA splicing, stability, and translation efficiency [49]. Two patients with LFS had an invasive lobular carcinoma, a histopathological type not previously reported in other series assessing the histological features of breast neoplasms in patients with this syndrome. All HER2-positive patients had characteristics that have been widely described in the literature [20, 45, 46, 50].

Different studies have reported *TP53* mutation frequencies from 3 to 8% in patients with BC aged younger than 30 years or in patients aged 30–39 with BC with family histories of LFS-associated cancers [28, 40, 41]. De novo *TP53* mutations have been reported in 7 to 20% of BC cases [42, 51]. It is rare to find *TP53* mutations in patients with BC older than 50 [11, 24, 52]. Lung cancer was the most frequently reported tumor in the family members of our patients with *TP53* gene mutations; until the 2009 proposal of the Chompret criteria, lung cancer was included among the tumors related to LFS [22].

Germline mutations in the *TP53* gene may explain a substantial percentage of BC in young Mexican women. In routine clinical practice, it is essential to recognize the relevance of familial history in identifying this group of high-risk patients. All patients in whom pathogenic variants were detected had a family history suggestive of a hereditary predisposition to cancer [10, 20, 53–57]. Incomplete penetrance is a crucial factor to consider in the assessment of LFS, even more so if the family history of malignancy comes from the paternal side.

The Mexican National Guidelines of Breast Cancer recommends offering molecular tests for the suspected gene/syndrome in women with BC and at high risk for hereditary cancer predisposition. Regarding multigene panels for hereditary cancer, the guidelines suggest that they should be ordered only by a geneticist with experience in hereditary cancer risk assessment who recognizes the limitations of the panels, due to the lack of clinical guidelines and a high percentage of variants of uncertain clinical significance that can be obtained by performing these studies.

Our project is one of the first that involves the analysis of genes predisposing women to BC other than *BRCA*, which is information that will help us in the clinical context of timely detection, prevention, and even risk modification. Importantly, none of these mutations or other pathological variants in *TP53* were found in the healthy control group.

## Conclusions

Knowledge about the prevalence of germline *TP53* mutations in young women with BC in the Mexican population will facilitate the implementation of specialized clinical programs, which will directly impact the prognosis of this particular group of patients and their families.

### Perspectives

The importance of distinguishing between an isolated tumor not related to germline mutations and cancer predisposition syndromes lies in the impact that this exerts on the family, at the economic and social levels for the health system (it is less costly to treat early-stage cancer), and at the social level, avoiding death of young patients who frequently have small children.

The identification of a mutation in a family makes it feasible to analyze at-risk relatives, potentially allowing early detection and prevention in carriers [58, 59].

### Study limitations

One limitation of our study is the possible recruitment bias because our sample was obtained by convenience from a large cancer risk predisposition cohort. An increase in the sample size could yield a more accurate estimation of the prevalence of mutations in *TP53*, as well as the identification of other novel putative mutations among this group of patients.

## Abbreviations

BC: Breast cancer
IARC: International Agency for Research on Cancer
INCan: Instituto Nacional de Cancerología
LFS: Li-Fraumeni Syndrome
NGS: Next-generation sequencing
OMIM: Online Mendelian Inheritance in Man
U.S.: United States of America
VUS: Variant of uncertain significance
